# Dynamic Response of Ion Transport in Nanoconfined Electrolytes

**DOI:** 10.1021/acs.nanolett.3c02560

**Published:** 2023-10-17

**Authors:** Zengming Zhang, Chenkun Li, Jianbo Zhang, Michael Eikerling, Jun Huang

**Affiliations:** † IEK-13, Institute of Energy and Climate Research, Forschungszentrum Jülich GmbH, 52425 Jülich, Germany; ‡ School of Vehicle and Mobility, State Key Laboratory of Automotive Safety and Energy, Tsinghua University, Beijing 100084, China; § Chair of Theory and Computation of Energy Materials, Faculty of Georesources and Materials Engineering, RWTH Aachen University, 52062 Aachen, Germany; ∥ Theory of Electrocatalytic Interfaces, Faculty of Georesources and Materials Engineering, RWTH Aachen University, 52062 Aachen, Germany

**Keywords:** ion transport, nanoconfinement, nonlinear Poisson−Nernst−Planck, solid electrolyte, impedance

## Abstract

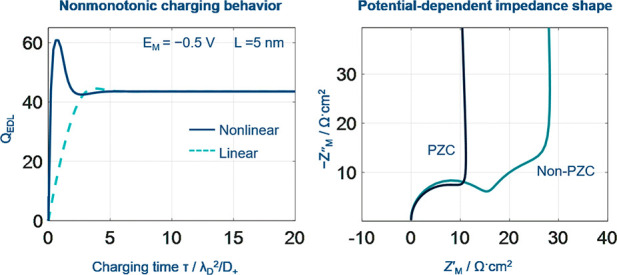

Ion transport in nanoconfined electrolytes exhibits nonlinear effects caused by large driving forces and pronounced boundary effects. An improved understanding of these impacts is urgently needed to guide the design of key components of the electrochemical energy systems. Herein, we employ a nonlinear Poisson–Nernst–Planck theory to describe ion transport in nanoconfined electrolytes coupled with two sets of boundary conditions to mimic different cell configurations in experiments. A peculiar nonmonotonic charging behavior is discovered when the electrolyte is placed between a blocking electrode and an electrolyte reservoir, while normal monotonic behaviors are seen when the electrolyte is placed between two blocking electrodes. We reveal that impedance shapes depend on the definition of surface charge and the electrode potential. Particularly, an additional arc can emerge in the intermediate-frequency range at potentials away from the potential of zero charge. The obtained insights are instrumental to experimental characterization of ion transport in nanoconfined electrolytes.

Ion transport is a fundamental process and sometimes the rate-determining step in electrochemical energy technologies, such as lithium-ion batteries. In the bulk phase, ion transport is often described using Fick’s law with the ion flux being proportional to the concentration gradient.^1−4^ Near a charged interface, ion flux driven by the spatially varying electrical field cannot be neglected, and Poisson–Nernst–Planck (PNP) theory is often used in this context.^5−8^ Modified PNP theories have been developed for ion transport in concentrated solutions where the finite size of ions and short-range correlations between ions are important.^9−15^ In addition, dynamic density functional theory provides a unified framework to describe ion transport in complex fluids.^16−18^ In spite of the formal differences, these theories assume that the ion flux is linear with respect to the driving force, viz., the gradient of the electrochemical potential; different treatments lead to different expressions of the driving force and diffusion coefficient.

In general, the reaction flux is an exponential function of the driving force. One example is the Butler–Volmer equation for charge transfer at the interface between two phases. Ion transport could be viewed as a chain of ion-vacancy coupled charge transfer reactions.^19,20^ This view leads to a Butler–Volmer-type equation for ion transport in which the ion flux is an exponential function of the electrochemical potential gradient of the examined ion, which was earlier developed by Riess and Maier.^21^ When the gradient of the electrochemical potential is small, the exponential function can be linearized, and a PNP-type equation is retrieved.^22^ This linear approximation is valid for ion transport in the bulk phase as well as in thick electric double layers (EDL). However, one would expect it to become increasingly problematic when the length scale reduces to the order of Debye length, namely, when ion transport occurs in the presence of large electric fields (above 100 kV/cm).^23−27^

In this paper, we study ion transport in an electrolyte, be it solid or liquid, under nanoconfinement where the characteristic length is comparable to the Debye length. Under these conditions, nonlinear ion transport kinetics has been observed.^28−30^ A nonlinear PNP theory is employed for this purpose. To treat different boundary conditions found in practical systems, two types of cells, including single-blocking open cells (SBOC) and double-blocking closed cells (DBCC), are analyzed in detail in both time and frequency space. Though the models are simple and not new, several unexpected behaviors are observed, including nonmonotonic EDL charging behaviors in the time space for the SBOC, the dependency of impedance shape on the definition of the EDL charge, and an additional impedance arc at potentials deviating from the potential of zero charge (PZC) for the SBOC. The model for the DBCC case is further employed to interpret experimental impedance data of ion transport in solid electrolytes. We also briefly discuss the influence of charge transfer reactions on the results, leaving a detailed analysis for a future study.

We start with a 1D model with an *ideally blocking* electrode and an electrolyte solution consisting of a binary symmetric electrolyte with ions of the same size. Within a primitive picture, the solvent is treated as a dielectric continuum with a constant permittivity ε_*S*_. Considering the finite size of ions, we introduce a finite space between the electrode surface and the edge of the electrolyte phase, denoted the Helmholtz plane (HP), of which the permittivity ε_HP_ is much lower than ε_S_.^31^ Nanoconfined electrolytes can have various boundary conditions in electrochemical energy devices. In metal-ion batteries, the solid–electrolyte interphase (SEI) can be mimicked as a nanoconfined solid electrolyte sandwiched between an electrode and a bulk electrolyte solution.^32−34^ Because the EDL can exchange particles with the electrolyte solution, we refer to it as a single-blocking open cell (SBOC), as illustrated in Figure 1. In measurements of the ion conductivity of solid electrolytes for solid-state batteries,^35−37^ the solid electrolyte is sandwiched between two blocking metals, and this case corresponds to a double-blocking closed cell (DBCC).

**Figure 1:**
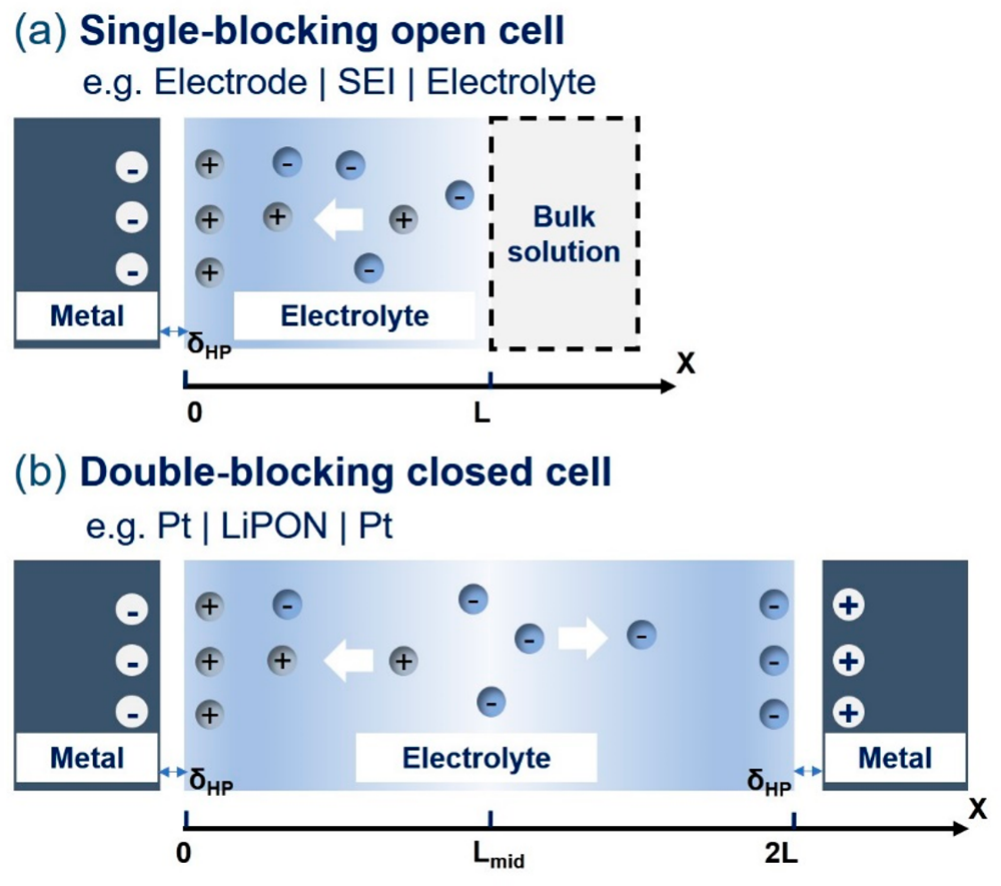
Schematic diagram of the two types of models. (a) Single-blocking open cell with one side in contact with a blocking electrode and the other side connected to a reservoir of electrolyte solution. (b) Double-blocking closed cell with an electrolyte solution confined between two blocking electrodes. The solution consists of a binary symmetric electrolyte with ions of the same size.

Ion transport in nanoconfined electrolytes is described by a modified PNP theory,^20,38^ which in the dimensionless form is given by
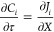
1
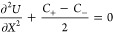
2where *C*_*i*_ is the ion concentration referenced to the bulk concentration *c*_0_^*i*^, the subscript *i* representing cations (+) or anions (−), *X* is the spatial coordinate normalized by the Debye length , τ = *tD*_+_/λ_D_^2^ is the dimensionless time, *D*_*i*_ is the diffusion coefficient of species *i*, and *U* = *F*ϕ/*RT* is the dimensionless potential; other symbols have their usual meaning.

The flux term is given by^19^
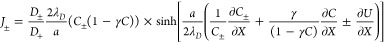
3with γ = *a*^3^*c*_0_*N*_A_ being the volume fraction of all ion in the bulk and *C* = *C*_+_ + *C*_–_ the total ion concentration. This nonlinear PNP equation can be reduced back to the linear PNP when the system remains in near-equilibrium because sinh ζ ≈ ζ for ζ ≪ 1:^19,20^
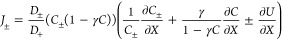
4At the left boundary, designated at the HP, *X* = 0, the flux vanishes for both SBOC and DBCC cases:
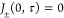
5As there is no extra charge in the space between the metal electrode surface and the HP, the potential distribution is linear in this region,^39,40^
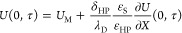
6where *U*_M_ is the surface potential applied on the left metal electrode.

The right boundary condition is contingent on the type of cell. For the SBOC, the nanoconfined electrolyte is connected with a constant-potential reservoir of electrolyte at *X* = *L*, namely
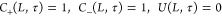
7meaning that ion concentrations assume their bulk values and the electric potential there serves as the reference.

For the DBCC, the right boundary at *X* = 2*L* has a zero ion flux
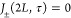
8and the electrode potential satisfies
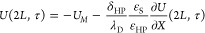
9with −*U*_M_ being the applied potential on the right metal.

Before applying the potential perturbation, we find the electrolyte solution in uniform concentrations and zero electric potential for both SBOC and DBCC, namely
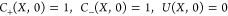
10We employ the present model to explore the charging dynamics of nanoconfined electrolytes, described in terms of the EDL charge density as a function of time. Two definitions of EDL charge exist in the literature,^16,41,42^ as shown in Figure S1, including the total diffuse charge
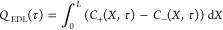
11and the electrode surface charge
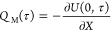
12These two charges are equivalent, if the electric field vanishes at *X* = *L*. Note that *Q*_M_(τ) does not start from zero because the initial electric field at *X* = 0 has a nonzero value, as shown in Figure S2. In the following, we use consistently the total diffuse charge *Q*_EDL_(τ) to describe the charging dynamics of the SBOC in Figure 2, and we provide results in terms of *Q*_M_(τ) in Figure S3. The charging dynamics of EDL has been widely studied.^43−45^ Conventionally, it can be divided into a fast process with a time constant of τ_RC_ = λ_D_*L*/*D*_+_ and a slow process with a time constant of τ_D_ = *L*^2^/*D*_+_.^6,40,46,47^ Such two-stage charging behaviors are observed for “thick”, namely, *L* ≫ λ_D_, electrolyte films, which are well described by the linear PNP theory. The charging dynamics of the DBCC given in Figure S4a show a similar charging behavior.

**Figure 2:**
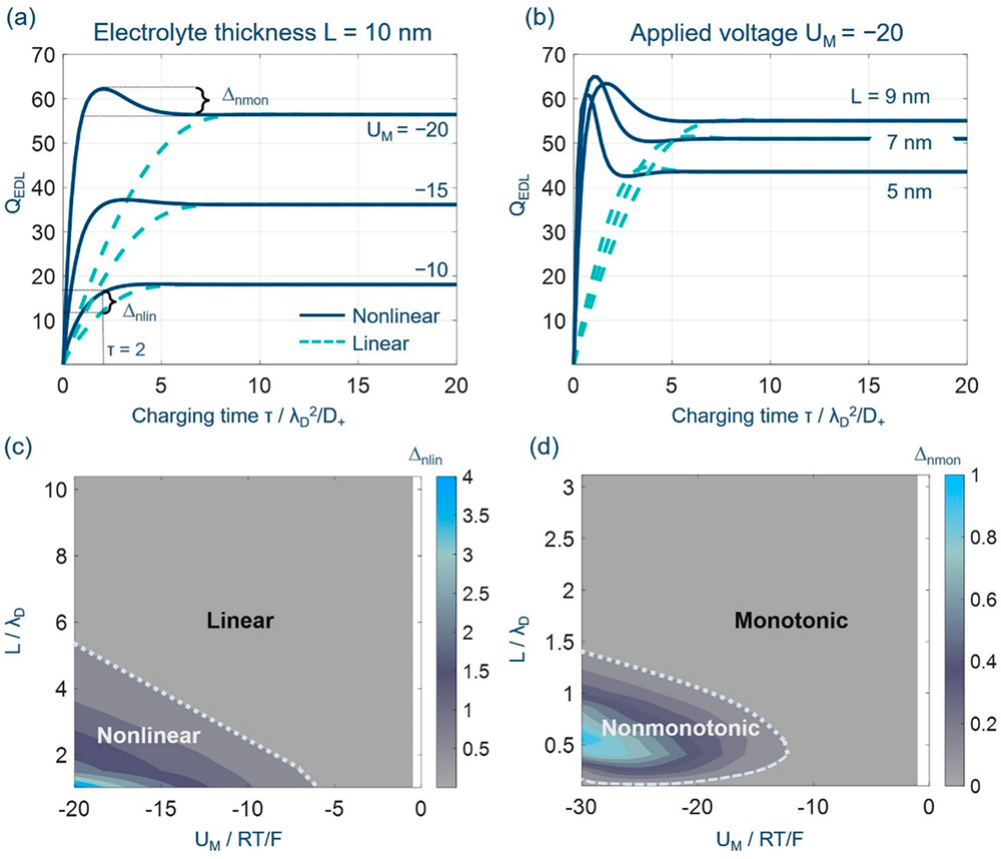
Charging dynamics of nanoconfined electrolytes in terms of the total diffuse charge *Q*_EDL_(τ) for the nonlinear (solid line) and linear PNP theory (dashed line) at (a) different applied voltages and (b) electrolyte thicknesses for the single-blocking open cells. (c) Regime of nonlinearity of the PNP theory. (d) Regime of nonmonotonic EDL charging dynamics. Model parameters are *c*_0_ = 1 × 10^–3^ mol L^–1^, *D*_±_ = 1 × 10^–11^ m^2^ s^–1^, and δ_HP_ = 0.3 nm, and the corresponding references values are λ_D_ ≈ 9.63 nm, *t*_ref_ = λ_D_^2^/*D*_+_ = 9.27 × 10^–7^ s, and *U*_ref_ = RT/F = 25 mV.

The nonlinear PNP theory differs from the linear PNP in two aspects. On the one hand, the nanoconfined electrolyte described using the nonlinear PNP theory charges faster, as shown in Figure 2a, because the ion flux is larger under the same driving force since sinh ζ > ζ. On the other hand, the *Q*_EDL_(τ) exhibits more pronounced nonmonotonic charging behaviors when nonlinear PNP theory is used. In addition, the nonmonotonicity is more pronounced when the EDL is driven further away from equilibrium, namely, when *U*_M_ is more negative, for the SBOC (see Figure 2a). The same phenomenon exists for *Q*_M_(τ) in Figure S3.

Is this nonmonotonic charging dynamics unique for the nonlinear PNP theory? No, we observe nonmonotonic charging dynamics when *L* is reduced below 0.52λ_D_ (5 nm for the present case) even for the linear PNP theory, as shown in Figure 2b. This indicates that the nonmonotonic charging dynamics is caused not by nonlinearity but by nanoconfinement. To interpret the nonmonotonic charging curves, we track the time evolution of the net charge density (ρ = *C*_+_ – *C*_–_) for the linear PNP theory at *L* = 0.52λ_D_ and *U*_M_ = −20 in Figure S5. We notice that when τ ≤ 3.8, the curve of ρ ∼ *X* is elevated with increasing time, indicating that counterions are attracted to the electrode surface to form the diffuse layer.^48^ When τ > 3.8, the curve of ρ ∼ *X* gradually decays because the surface charge is already overscreened by the counterions; thus, the excess amount of counterions need to be balanced by co-ions in the diffuse layer. Therefore, the net charge density decreases until equilibrium is reached.^11^ In summary, the proposed model reveals an overscreening phenomenon in SBOCs induced by nanoconfinement.

The nonlinear and nonmonotonic effects depend on two key parameters: *L* and *U*_M_. Herein, we introduce two related descriptors, Δ_nlin_ and Δ_nmon_:
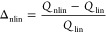
13which is the relative difference of *Q*_EDL_ between nonlinear PNP and linear PNP and
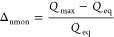
14which is the relative difference of *Q*_EDL_ between maximum and equilibrium values. Both ratios are functions of *U*_M_ and *L*, as shown in Figure 2c,d. In Figure 2c, we calculate Δ_nlin_ at a dimensionless time τ = 2 because the nonlinear effect is most pronounced in this range. Δ_nlin_ is larger at small *L* and large |*U*_M_|, and Δ_nlin_ is greater than 1 when *L* < 8.4λ_D_ (52 nm) and |*U*_M_| > 6.1 (0.15 V), namely, when the electric field *E* > 30 kV/cm. The electric field in the SEI has been estimated to be about >50 kV/cm, using nonlinear conductivity spectroscopy.^23,49^ Therefore, the nonlinear PNP theory is more accurate to describe ion transport in this case. In Figure 2d, Δ_nmon_ is greater than 0.2 when *L* < 1.4λ_D_ (13 nm) and |*U*_M_| > 12.5 (0.31 V), suggesting that nonmonotonic dynamic charging is more pronounced under such conditions. In other words, nonmonotonic charging behavior is enhanced in nanoconfinement with a higher electric field.

Electrochemical impedance spectroscopy (EIS) allows analysis of ion transport in a wide frequency range. The EIS responses for SBOC and DBCC are solved analytically at PZC^50,51^ (see technical details in Supplementary Note 5) and numerically at other potentials following the method of refs (52 and 53). There is no difference between the linear and nonlinear PNP theory in the EIS response because the sinusoidal potential is a small perturbation signal (see Figure S7). Contrary to the time-domain results, EIS calculated from two definitions of EDL charge are nontrivially different. In this section, we describe the EIS response of the SBOC, and we provide the results of the DBCC in Figure S8.

At the PZC, namely, *U*_M_^dc^ = 0, the impedance based on *Q*_EDL_ is analytically expressed as
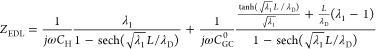
15where *C*_H_ is the Helmholtz capacitance, , *C*_GC_^0^ the Gouy–Chapman capacitance at PZC, , and . With asymptotical analysis provided in Supplementary Note 5, in the low-frequency range, eq 15 is asymptotic to
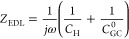
16a capacitive behavior corresponding to the equilibrium EDL capacitance. In the high-frequency range, eq 15 is asymptotic to
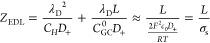
17a pure resistance behavior, where  is the electrical conductivity of the bulk electrolyte.

The impedance response from *Q*_M_ at the PZC is analytically obtained as
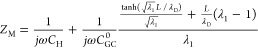
18

In the low-frequency range, eq 18 asymptotically approaches
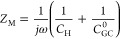
19which is the same as *Z*_EDL_ in the low-frequency range. In the high-frequency range, eq 18 approaches
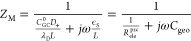
20where  is the electrolyte resistance at the PZC and  the geometric capacitance of the electrolyte.

In contrast to a pure resistor given by *Z*_EDL_, a semicircle is expected in the high-frequency range for *Z*_M_. Consistent with the above theoretical analysis, the EIS calculated based on *Q*_EDL_ shows a nearly vertical line, the EIS calculated based on *Q*_M_ shows a semicircle in high-frequency range followed by a vertical line in low-frequency range (see Figure 3a). The agreement between analytical and numerical results at δ_HP_ = 0 confirms the accuracy of the numerical method when HP is not considered. In Figure 3b, the influence of the HP on analytical and numerical solutions at the PZC is examined. The existence of the HP brings about an anomalous semicircle in the second quadrant for the numerical results in the dashed lines. This anomalous feature is a numerical artifact because it disappears in the analytical results in the solid lines. Neglecting the HP, we find the numerical results are converged to the analytical results, and the mere difference is in the length of the low-frequency vertical line, due to the change of the EDL capacitance (cf. eq 19). Hence, we neglect the HP in subsequent analysis because our focus is put on the high-frequency semicircle.

**Figure 3:**
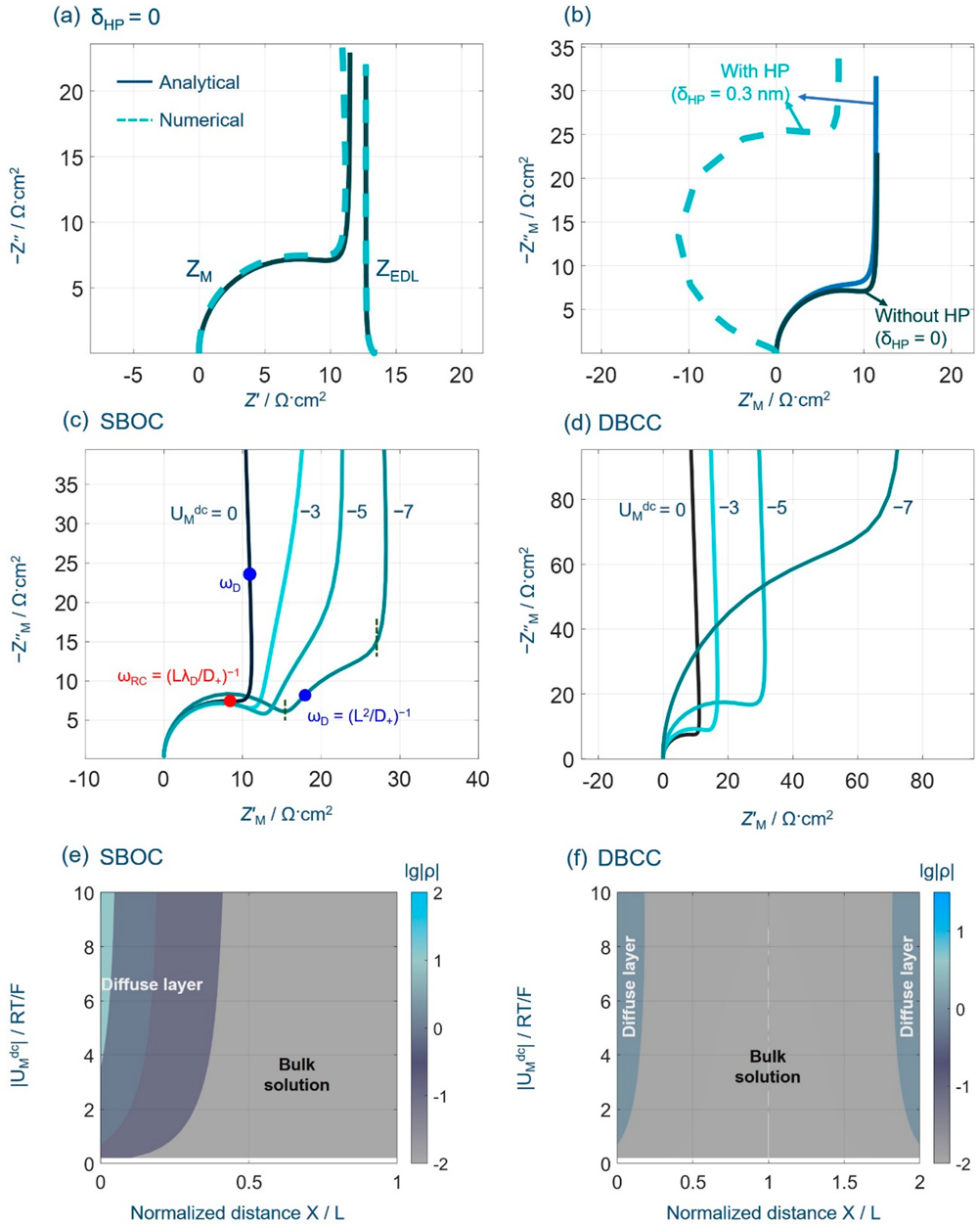
EIS of ion transport in the single-blocking open cells. (a) Comparison between the EIS calculated from the total diffuse charge, denoted as *Z*_EDL_, and that from the electrode surface charge, *Z*_M_, at the potential of zero charge, *U*_M_^dc^ = 0 and δ_HP_ = 0 nm. Analytical and numerical results are displayed as solid and dashed lines, respectively. (b) Influence of the Helmholtz plane (HP) on analytical (solid lines) and numerical (dashed lines) solutions of *Z*_M_. *Z*_M_ at different electrode potentials *U*_M_^dc^ for the (c) SBOC and (d) the DBCC at *L* = 100 nm and δ_HP_ = 0 nm. (e, f) Distribution of the net charge density at different *U*_M_^dc^’s for the SBOC and the DBCC, respectively. Model parameters are *c*_0_ = 1 × 10^–3^ mol L^–1^, *D*_±_ = 1 × 10^–11^ m^2^ s^–1^, *E*_M_ = 2.5 × 10^–3^ sin ω*t* V, and *E*_eq_ = *E*_pzc_ = 0. The frequency range is 1 × 10^6^–1 × 10^–1^ Hz.

When the potential deviates from the PZC, namely, *U*_M_^dc^ ≠ 0, a newly tilted line can be observed in the intermediate frequency of *Z*_M_ in Figure 3c. This can be attributed to the finite-rate ion transport in the inhomogeneous electrolyte featuring a time scale of τ_D_ = (ω_D_)^−1^ = *L*^2^/*D*_+_, leading to frequency dispersion of the double-layer capacitance. This frequency-dispersion phenomenon is observed only in the SBOC, not in the DBCC of which the EIS is given in Figure 3d. The reason is that the diffuse layer is more pronounced in the SBOC than in the DBCC when *U*_M_^dc^ ≠ 0, as shown in Figure 3e,f. Ion transport in the whole of the electrolyte features a time constant of τ_RC_ = (ω_RC_)^−1^ = λ_D_*L*/*D*_+_.

Katayama et al. measured the EIS of Ni/LiPON/Li at several electrode potentials.^54^ The Nyquist plots show a semicircle in the high-frequency range, followed by a nearly vertical line; they found that the diameter of the semicircle derived from the LiPON thin film increases with increasing the electrode potential, consistent with the trend of *Z*_M_ of DBCC in Figure 3d. The potential dependence of the impedance of Ni/LiPON/Li is reproduced in Figure 4a. Figure 4b shows the dependence of the electrolyte resistance, *R*_ele_, defined as *R*_ele_ = *Z*_M_^′^ (ω → 0), of the DBCC on electric potential *U*_M_^dc^. The model-based result in Figure 4b is calculated using the parameters from refs (37, 54, and 55) where *c*_0_ = 0.25 M, *D*_+_ = 2 × 10^–12^ m^2^ s^–1^, ϵ_s_ = ϵ_LiPON_ = 16.6ε_0_, and 2*L* = 760 nm. Increasing the applied potential, the electrolyte resistance *R*_LiPON_ increases gradually due to the decreasing ion concentration at the middle plane, *C*_+,mid_^eq^, as shown in Figure 4c,d. To save the calculation time, we calculate *C*_+,mid_^eq^ using 2*L* = 200 nm. Because *C*_+,mid_^eq^ is uniformly distributed, increasing the electrolyte thickness to 760 nm will not affect the results. The steady-state cation concentration *C*_+,mid_^eq^ is decreased at a more negative *U*_M_^dc^. The relationship between *C*_mid_^eq^ and *U*_M_^dc^ can be described by an approximate analytical expression originally given in ref (56).
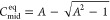
21where
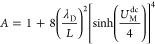
22

**Figure 4:**
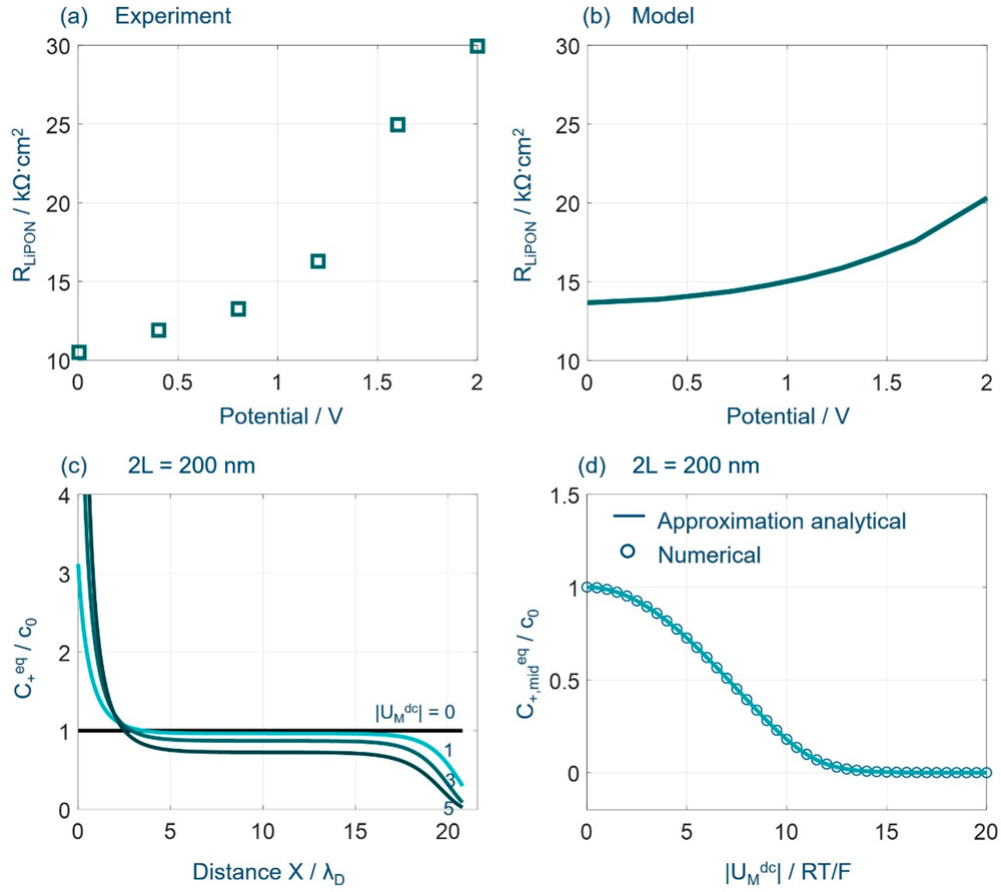
EIS of ion transport in the double-blocking closed cells. (a, b) Comparison between model and experimental results of the EIS response at different potentials. Experimental data were reported by Katayama et al. in Ni/LiPON/Li.^54^ The model-based result is calculated using *c*_0_ = 0.25 M, *D*_+_ = 2 × 10^–12^ m^2^ s^–1^, ϵ_s_ = ϵ_LiPON_ = 16.6ε_0_, and 2*L* = 760 nm. (c) shows the steady-state distribution of cation concentration at different applied voltages, and (d) the steady-state cation concentration at the middle plane as a function of applied voltage. Analytical and numerical results are displayed as solid and dashed lines, respectively. Model parameters are *c*_0_ = 1 × 10^–3^ mol L^–1^, *D*_±_ = 1 × 10^–11^ m^2^ s^–1^, 2*L* = 200 nm, and δ_HP_ = 0 nm.

Figure 4 indicates that the analytical solution captures numerical results. Experimental values are overall larger than model-based values, which could be attributed to the surface roughness of the metal electrode.^32,33,57^

So far, our analysis has been focused on blocking electrodes. As practical situations usually involve reactive, nonblocking electrodes, one may wonder if the insights collected on the blocking electrodes also apply for nonblocking electrodes. This question is briefly touched upon below.

Specifically, two more cases involving nonblocking electrodes are considered, including a single reactive open cell with the left side in contact with a nonblocking electrode and the right side connected to a reservoir of electrolyte solution, and a single blocking closed cell with the left side in contact with a blocking electrode and the right side connected to a nonblocking electrode (see Supplementary Notes 5 and 9).

For the case of single reactive open cell, the nonlinear and nonmonotonic effects are also observed, and the quantitative difference is that *Q*_EDL_(τ) decreases at larger rate constant *k*_0,ct_. This is because the metal deposition reaction consumes cations, thus lowering *Q*_EDL_. The regimes of nonlinearity of the PNP theory and nonmonotonic EDL charging dynamics are basically the same as for the case of SBOC. Therefore, we conclude that the main conclusions previously drawn for the single blocking open cell are also applicable to a reactive electrode. For the case of single blocking closed cell, we notice that the EIS consists of two semicircles in high- and intermediate-frequency range and a vertical line in the low-frequency range. With an increasing rate constant *k*_0,ct_, the intermediate-frequency semicircle associated with the charging transfer decreases. The high-frequency semicircle corresponds to the electrolyte resistance in parallel with the geometric capacitance, and a vertical line in low-frequency range corresponds to the equilibrium EDL capacitance. Therefore, a nonblocking metal on the right side will bring forth a new semicircle attributed to the charging transfer reaction in the intermediate-frequency range.

In summary, nonlinear-PNP theory has been employed to describe ion transport in nanoconfined electrolytes in single-blocking open cell (SBOC) and double-blocking closed cell (DBCC) configurations. The SBOC shows a surprising nonmonotonic double-layer charging behavior. When the EDL charge refers to the total ionic charge in the diffuse layer, the EIS shows a nearly vertical line. When the EDL charge refers to the electrode surface charge, the EIS shows a semicircle in the high-frequency range and a vertical line in the low-frequency range. An additional impedance arc in the moderate to low frequency range is observed only for the SBOC at potentials deviating from the potential of zero charge. The high-frequency semicircle represents the electrolyte resistance in parallel with the geometric capacitance of the electrolyte. The tilted line at intermediate frequencies represents the ion transport in the inhomogeneous electrolyte, leading to the frequency dispersion of the double-layer capacitance. The low-frequency vertical line is associated with the equilibrium double-layer capacitance. Experimental data of ion transport in a solid electrolyte are interpreted using the DBCC model. We also briefly discussed the influence of charge transfer reactions on the results. For the case of a single reactive open cell, the time-domain charging dynamic behaviors are qualitatively the same as in the SBOC case. In frequency space, a nonblocking metal on the right side will bring forth a new semicircle attributed to the charging transfer reaction in the intermediate-frequency range. A more detailed analysis of reactive, nonblocking electrodes will be reported in the future.

## Supporting Information Available

Comparison between *Q*_EDL_ and *Q*_M_ in time space; double layer charging dynamics in terms of *Q*_M_ for the SBOC; comparison of *Q*_EDL_ between the DBCC and the SBOC; time evolution of net charge density distribution in the SBOC; double layer charging dynamics in terms of *Q*_EDL_ for the single reactive open cell; analytical solution of EIS at PZC; comparison of EIS between linear PNP and nonlinear PNP theory for the SBOC; EIS response of the DBCC; EIS response of the single blocking closed cell (PDF)
