# Supplementary material for: Dynamic Response of Ion Transport in Nanoconfined Electrolytes
Source: Nano Lett. 2023 Oct 17;23(23):10703–9. doi: 10.1021/acs.nanolett.3c02560 (PMC10722536; doi:10.1021/acs.nanolett.3c02560)
Supplement: Supplementary file 1 — nl3c02560_si_001.pdf [file nl3c02560_si_001.pdf]

Supporting Information for

Dynamic Response of Ion Transport in Nanoconfined Electrolytes

Zengming Zhang[1], Chenkun Li[1], Jianbo Zhang[2], Michael Eikerling[1,3], Jun Huang[1,4*]

1 IEK-13, Institute of Energy and Climate Research, Forschungszentrum Jülich GmbH, 52425, Jülich, Germany

2 School of Vehicle and Mobility, State Key Laboratory of Automotive Safety and Energy, Tsinghua University, Beijing 100084, China

3 Chair of Theory and Computation of Energy Materials, Faculty of Georesources and Materials Engineering, RWTH Aachen University, 52062 Aachen, Germany

4 Theory of Electrocatalytic Interfaces, Faculty of Georesources and Materials Engineering, RWTH Aachen University, 52062 Aachen, Germany

** Corresponding author, e-mail: ju.huang@fz-juelich.de*

1. Comparison between Q_{EDL} and Q_{M} in time space

There are two common definitions of the EDL charge[1, 2], including the total diffuse charge $Q_{\mathrm{EDL}}(\tau)$, and the electrode surface charge $Q_{\mathrm{M}}(\tau)$, as shown in FIG S1. $Q_{\mathrm{EDL}}(\tau)$ is calculated from the integration of the net ionic charge from the Helmholtz plane to the middle plane, while $Q_{\mathrm{M}}(\tau)$ is calculated by the Gauss's law. Taking the double-blocking closed cells (DBCC) for example, we compare the dimensionless $Q_{\mathrm{EDL}}(\tau)$ (solid line) and $Q_{\mathrm{M}}(\tau)$ (dot-dashed line) at an applied voltage $U_{\mathrm{M}} = -1$ (25 mV) for an electrolyte film with a thickness of $2L = 10.4\lambda_{\mathrm{D}}$ (100 nm for the 1mM solution), as shown in FIG S2. These two EDL charging dynamic curves show the same trend, and eventually increase to the same equilibrium value because the electric field is close to zero at the middle plane. It should be noted that the initial value of $Q_{\mathrm{M}}(\tau)$ does not start from zero because the initial electric field departs from zero.

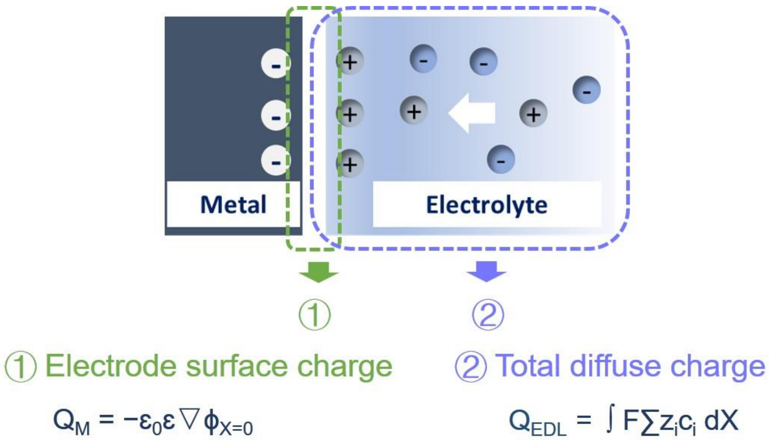

FIG S1. Two definitions of EDL charge, the electrode surface charge $Q_{\mathrm{M}}(\tau)$ by Gauss's law and total diffuse charge $Q_{\mathrm{EDL}}(\tau)$ by integrating the ionic charge from the Helmholtz plane to the middle plane.

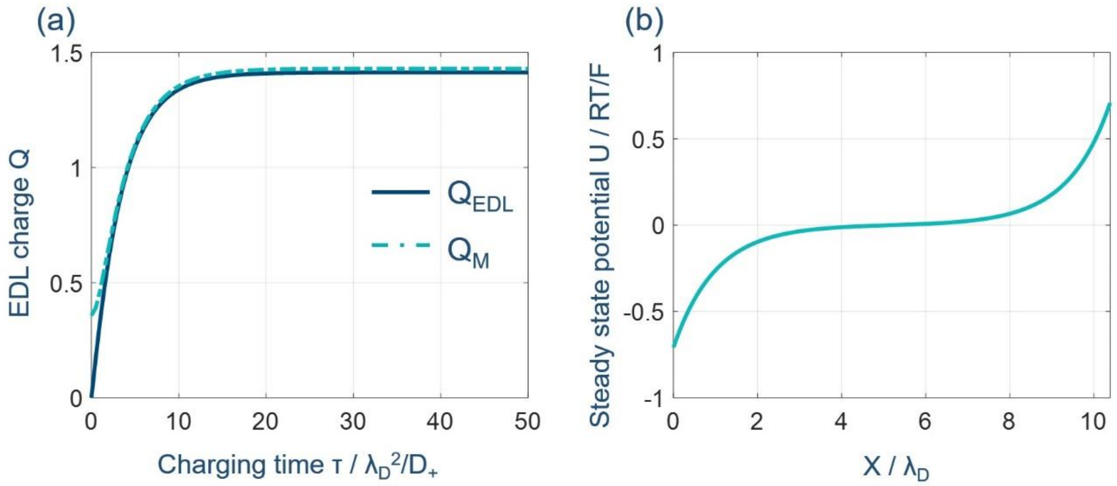

FIG S2. Taking the double-blocking closed cell as an example, we compare Q_{EDL} and Q_{M} in terms of EDL charging behavior in (a) and electric potential distribution at a steady state in (b). Model parameters are $c_0 = 1 \times 10^{-3}\ \mathrm{mol\ L^{-1}}, D_{\pm} = 1 \times 10^{-11}\ \mathrm{m^2\ s^{-1}}, \delta_{\mathrm{HP}} = 0.3\ \mathrm{nm}, U_{\mathrm{M}} = -1, 2L = 100\ \mathrm{nm}$, and the corresponding reference values are $\lambda_{\mathrm{D}} \approx 9.63\ \mathrm{nm}, t_{\mathrm{ref}} = {\lambda_D}^2/D_{+} = 9.27 \times 10^{-7}\ \mathrm{s}, U_{\mathrm{ref}} = RT/F = 25\ \mathrm{mV}$.

2. Double layer charging dynamics in terms of Q_{M} for the SBOC

In FIG S3, we plot the double layer charging dynamics in terms of the electrode surface charge Q_{M}. The corresponding Q_{EDL} is shown in FIG (2) of the main text.

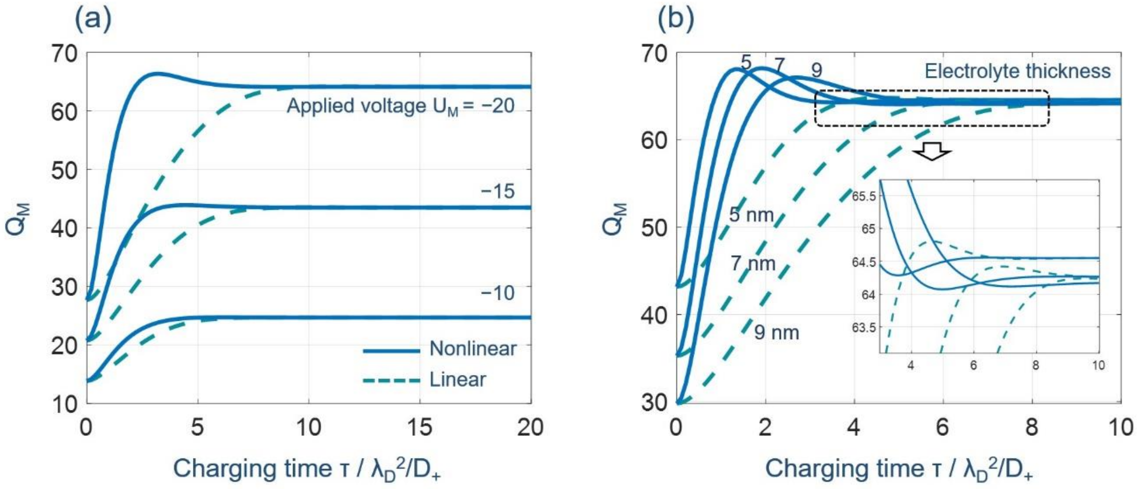

FIG S3. For the single-blocking open cells, we compare the time evolution of the electrode surface charge Q_{M} for the nonlinear (line) and linear (dash line) PNP theory at (a) different applied voltages and (b) thicknesses of the electrolyte, respectively. Model parameters are $c_0 = 1 \times 10^{-3}$ mol L^{-1}, $D_{\pm} = 1 \times 10^{-11}$ m^2 s^{-1}, $\delta_{\mathrm{HP}} = 0.3$ nm.

3. Comparison of Q_{EDL} between the DBCC and the SBOC

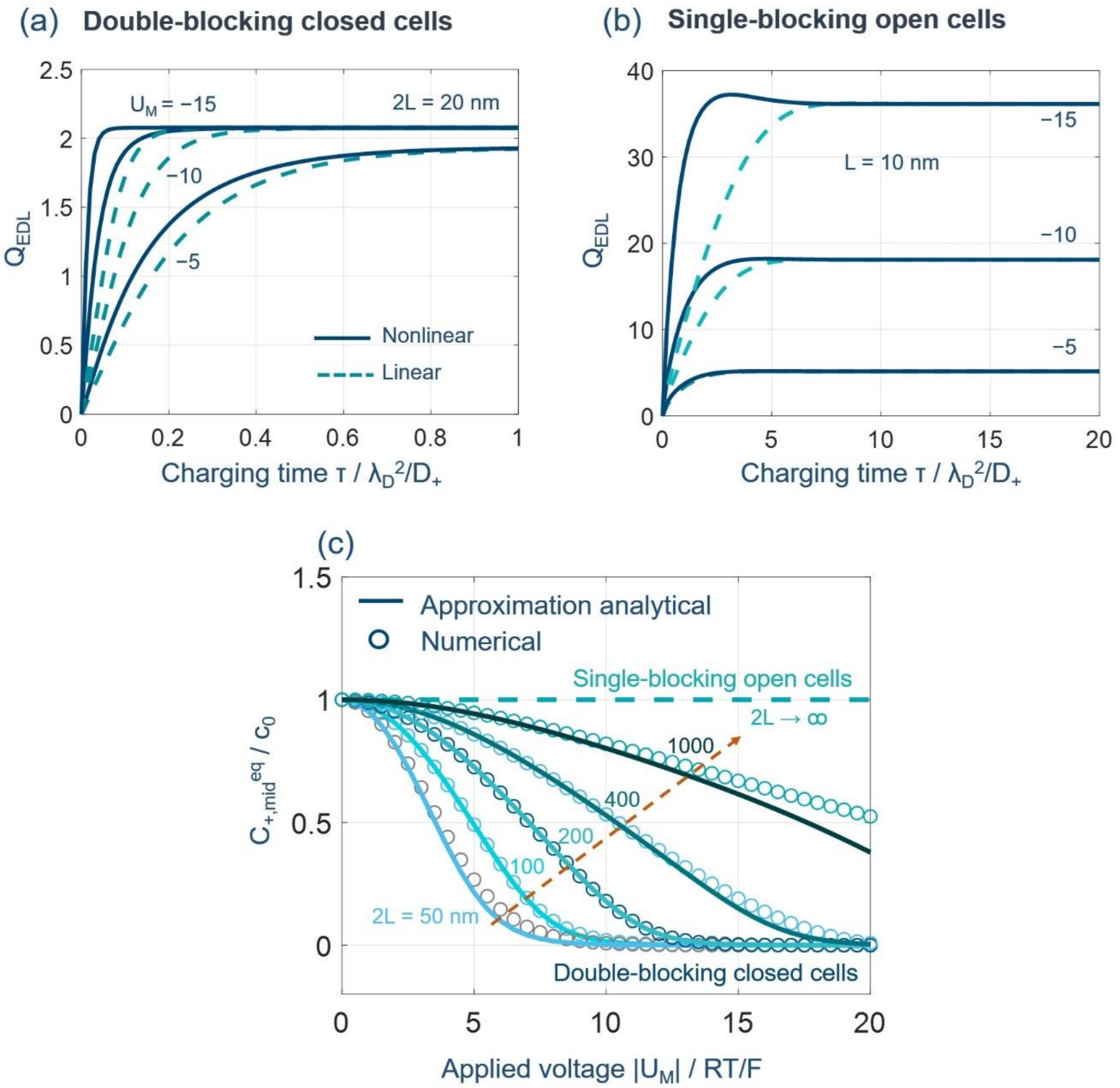

FIG S4. Comparison of Q_{EDL} between (a) the DBCC and (b) the SBOC at different U_{M}. (c) Comparison of $C_{+,\mathrm{mid}}^{\mathrm{eq}}$ of DBCC as function of U_{M} between the analytical (solid line) and the numerical (circle) results for different electrolyte film thicknesses. Model parameters are $c_0 = 1 \times 10^{-3}\ \mathrm{mol\ L^{-1}}, D_{\pm} = 1 \times 10^{-11}\ \mathrm{m^2\ s^{-1}}, \delta_{\mathrm{HP}} = 0.3\ \mathrm{nm}$.

In the main text, we have introduced the single-blocking open cells (SBOC) and double-blocking closed cell (DBCC). Here, we depict the double layer charging dynamics in terms of Q_{EDL} for the DBCC and the SBOC, as shown in FIG S4(a-b), respectively. Firstly, the EDL charges faster when the nonlinear PNP theory is used for both the DBCC and the SBOC, because it gives a larger ion flux under

the same driving force since $\sinh\zeta > \zeta$. Secondly, the equilibrium value of the total diffuse charge, $Q_{\mathrm{EDL}}^{\mathrm{eq}} = Q_{\mathrm{EDL}}(\infty)$, for the DBCC is much smaller than the SBOC. This is because the number of ions inside the DBCC is limited and kept constant, while the SBOC is an open system connected to a reservoir. Herein, FIG S4 (c) shows the steady state cation's concentration at the middle plane, $C_{+,\mathrm{mid}}^{\mathrm{eq}}$, for the DBCC as a function of U_{M} for different electrolyte film thicknesses. The $C_{+,\mathrm{mid}}^{\mathrm{eq}}$ can described by the following approximate analytical expression[3],

$$C_{+,\mathrm{mid}}^{\mathrm{eq}} = \mathrm{A} - \sqrt{\mathrm{A}^2 - 1}, \tag{S1}$$

where,

$$\mathrm{A} = 1 + 8\left(\frac{\lambda_D}{L}\right)^2 \left[\sinh\left(\frac{U_{\mathrm{HP}}^{\mathrm{eq}}}{4}\right)\right]^4, \tag{S2}$$

with λ_D being the Debye length, given by $\lambda_D = \sqrt{\varepsilon_{\mathrm{S}} RT/(2F^2 c_0)}$, L the electrolyte thickness from the Helmholtz plane (HP) to the middle plane, and $U_{\mathrm{HP}}^{\mathrm{eq}}$ the dimensionless steady-state potential at the HP, calculated using the Poisson-Boltzmann (PB) equation,

$$\frac{\partial^2 U}{\partial X^2} = \sinh U, \tag{S3}$$

coupled with the left boundary condition,

$$U_{\mathrm{HP}}^{\mathrm{eq}} = U_{\mathrm{M}} + \frac{\delta_{\mathrm{HP}}}{\lambda_D}\frac{\varepsilon_{\mathrm{S}}}{\varepsilon_{\mathrm{HP}}}\frac{\partial U}{\partial X}(X = 0). \tag{S4}$$

We obtain the relationship between the surface applied voltage U_{M} and the $U_{\mathrm{HP}}^{\mathrm{eq}}$,

$$U_{\mathrm{M}} = U_{\mathrm{HP}}^{\mathrm{eq}} - 2\frac{\delta_{\mathrm{HP}}}{\lambda_D}\frac{\varepsilon_S}{\varepsilon_{\mathrm{HP}}}\sinh\left(\frac{U_{\mathrm{HP}}^{\mathrm{eq}}}{2}\right). \tag{S5}$$

As expected, FIG S4 (c) shows that $C_{+,\mathrm{mid}}^{\mathrm{eq}}$ decreases with increasing U_{M}. This can be attributed to the fact that more counterions move to the electrode surface to counter the electrode surface charge. Increasing the electrolyte thickness will diminish the trend. The approximate analytical expression (S1) can explain this

phenomenon very well. When the electrolyte thickness increases to infinity, $2L \to \infty$, then $C_{+,\mathrm{mid}}^{\mathrm{eq}} = 1$, at any applied voltage, as shown in the dashed line in FIG S4 (c). It means that the DBCC is equivalent to the SBOC when $2L \to \infty$. Thirdly, compared with the DBCC, the SBOC shows a nonmonotonic charging behavior when the nonlinear PNP theory is used, which is discussed in the main text.

4. Time evolution of net charge density distribution in the SBOC

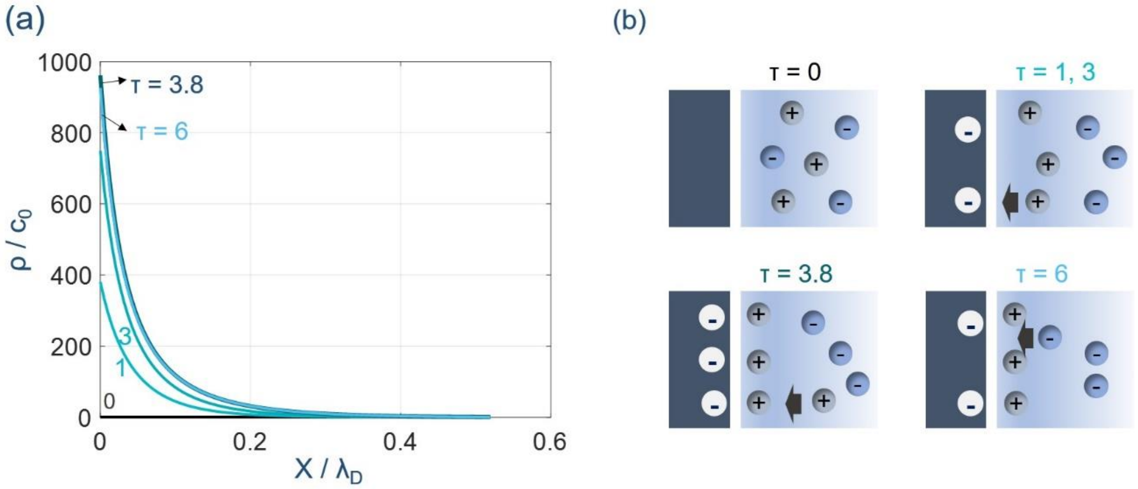

FIG S5. (a) Time evolution of the net charge density distribution in the SBOC; (b) Schematic diagram of physical processes. Model parameters are $L = 5\ \text{nm}, U_{\text{M}} = -20$, $c_0 = 1 \times 10^{-3}\ \text{mol L}^{-1}, D_{\pm} = 1 \times 10^{-11}\ \text{m}^2\ \text{s}^{-1}, \delta_{\text{HP}} = 0.3\ \text{nm}$.

FIG S5 (a) shows the time evolution of net charge density, $\rho = C_+ - C_-$, distribution in the SBOC at $L = 5\ \text{nm}, U_{\text{M}} = -20$. A schematic diagram of physical processes is shown in FIG S5 (b). This result is calculated by the linear PNP theory.

5. Double layer charging dynamics in terms of Q_{EDL} for the single reactive open cell

The influence of an electrochemical reaction on the EDL charging is examined by considering a single-reactive open cell, which involves a metal-deposition reaction at left boundary, $X = 0$, as show in FIG S6 (a),

$$\mathrm{M}^{+} + \mathrm{e}^{-} \leftrightarrow \mathrm{M} \tag{S6}$$

where M^{+} is the metal ion, and M the metal atom. The kinetics of this charge transfer reaction can be described using the Frumkin-Butler-Volmer (FBV) theory,

$$j_{ct} = k_{0,ct}\left(\exp\left(\frac{\alpha F\eta}{RT}\right) - C_{+,\mathrm{HP}}\exp\left(-\frac{(1-\alpha)F\eta}{RT}\right)\right) \tag{S7}$$

where $k_{0,ct}$ is the rate constant of charge transfer, $C_{+,\mathrm{HP}}$ the concentration of M^{+} at the Helmholtz plane (HP), α the charge transfer coefficient and $\eta = E_{\mathrm{M}} - \phi_{\mathrm{HP}} - E_{\mathrm{eq}}$ the overpotential with E_{eq} being the equilibrium potential. ϕ_{HP} the electric potential at the HP. Settings and parameters are the same as those in the SBOC case. The nonlinear and non-monotonic effects are also observed in this single-reactive open cell, and the mere difference is that $Q_{\mathrm{EDL}}(\tau)$ decreases at larger rate constant of charge transfer $k_{0,ct}$, as shown in FIG S6(b). This is because the metal deposition reaction consumes cations, thus lowering Q_{EDL}. Regime of nonlinearity of the PNP theory and non-monotonic EDL charging dynamics, as shown in FIG S6 (c-d), respectively, are basically the same as in the case of SBOC.

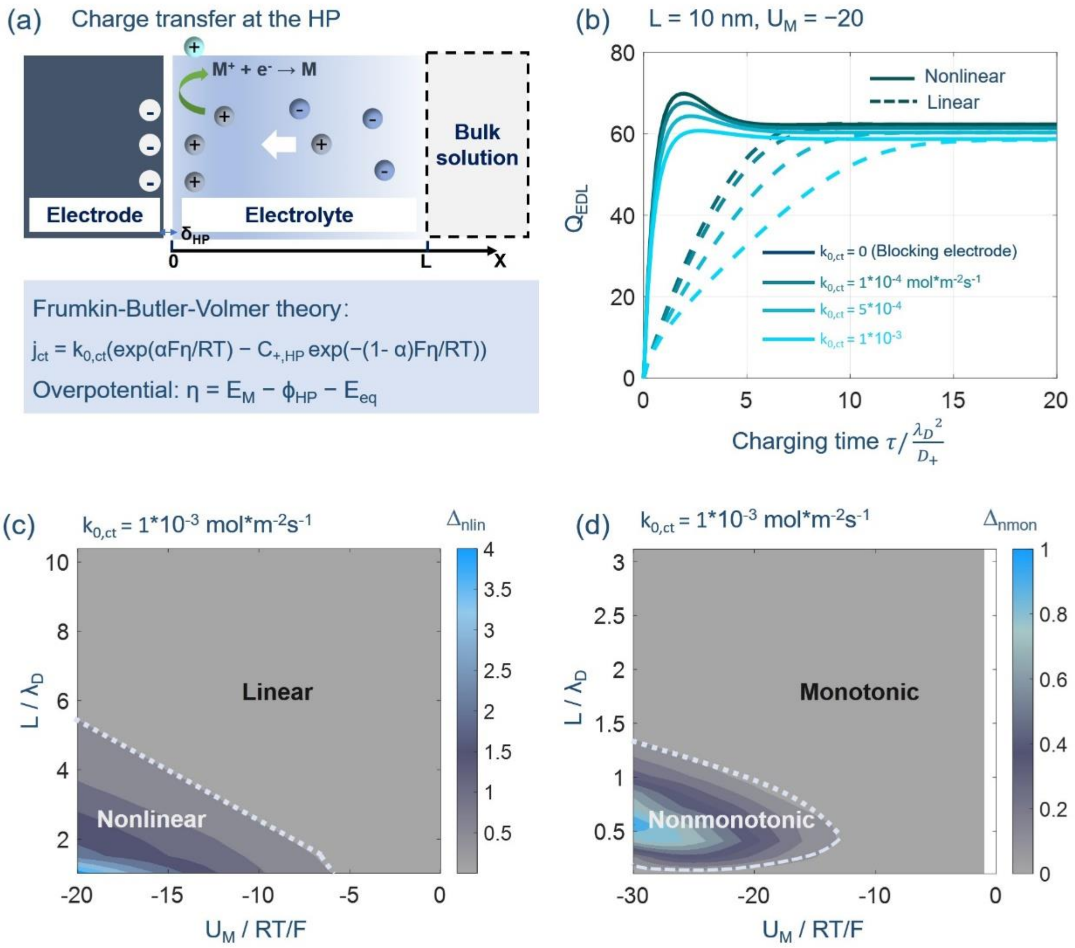

FIG S6. (a) Schematic diagram of the single-reactive open cell with one side in contact with a non-blocking electrode, and the other side connected to a reservoir of electrolyte solution; (b) The charging dynamics of nanoconfined electrolytes in terms of the total diffuse charge $Q_{\mathrm{EDL}}(\tau)$ for the nonlinear (line) and linear PNP theory (dash line) at different rate constants of the charge transfer reaction; (c) Regime of nonlinearity of the PNP theory. (d) Regime of non-monotonic EDL charging dynamics. Model parameters are $c_0 = 1 \times 10^{-3}\ \mathrm{mol\ L^{-1}}, D_{\pm} = 1 \times 10^{-11}\ \mathrm{m^2\ s^{-1}}, \delta_{\mathrm{HP}} = 0.3\ \mathrm{nm}$, and the corresponding references values are $\lambda_D \approx 9.63\ \mathrm{nm}, t_{ref} = {\lambda_D}^2/D_+ = 9.27 \times 10^{-7}\ \mathrm{s}, U_{ref} = RT/F = 25\ \mathrm{mV}$.

6. Analytical solution of EIS at PZC [4, 5]:

Herein, the ion size effect is ignored, using $\gamma = 0$, and the linear PNP equation can be simplified to,

$$\frac{\partial}{\partial \tau}\begin{bmatrix} C_+ \\ C_- \\ 0 \end{bmatrix} = \frac{\partial}{\partial X}\begin{Bmatrix} \frac{\partial C_+}{\partial X} + C_+\frac{\partial U}{\partial X} \\ \frac{\partial C_-}{\partial X} - C_-\frac{\partial U}{\partial X} \\ \frac{\partial U}{\partial X} \end{Bmatrix} + \begin{bmatrix} 0 \\ 0 \\ \frac{1}{2}(C_+ - C_-) \end{bmatrix}. \tag{S8}$$

Applying a perturbation to (S8), we obtain,

$$j\omega^{\mathrm{nd}}\begin{bmatrix} \tilde{C}_+ \\ \tilde{C}_- \\ 0 \end{bmatrix} = \frac{\partial}{\partial X}\begin{Bmatrix} \frac{\partial \tilde{C}_+}{\partial X} + C_+^0\frac{\partial \tilde{U}}{\partial X} + \tilde{C}_+\frac{\partial U^0}{\partial X} \\ \frac{\partial \tilde{C}_-}{\partial X} - C_-^0\frac{\partial \tilde{U}}{\partial X} - \tilde{C}_-\frac{\partial U^0}{\partial X} \\ \frac{\partial \tilde{U}}{\partial X} \end{Bmatrix} + \begin{bmatrix} 0 \\ 0 \\ \frac{1}{2}(\tilde{C}_+ - \tilde{C}_-) \end{bmatrix} \tag{S9}$$

where the over-tilde marks a quantity in the frequency domain and ω^{nd} is the dimensionless angular frequency with respect to $\frac{D_+}{\lambda_D^2}$. Due to the initial conditions $C_+^0 = C_-^0 = 1$, $U^0 = 0$, (S9) can be written as,

$$j\omega^{nd}\begin{bmatrix} \tilde{C}_+ \\ \tilde{C}_- \\ 0 \end{bmatrix} = \frac{\partial}{\partial X}\begin{Bmatrix} \frac{\partial \tilde{C}_+}{\partial X} + \frac{\partial \tilde{U}}{\partial X} \\ \frac{\partial \tilde{C}_-}{\partial X} - \frac{\partial \tilde{U}}{\partial X} \\ \frac{\partial \tilde{U}}{\partial X} \end{Bmatrix} + \begin{bmatrix} 0 \\ 0 \\ \frac{1}{2}(\tilde{C}_+ - \tilde{C}_-) \end{bmatrix} \tag{S10}$$

The (S10) can be solved,

$$\tilde{C}_{+} = -y_1 + y_2$$

$$y_1 = \alpha_1 \sinh\left(\sqrt{\lambda_1} X\right) + \alpha_2 \cosh\left(\sqrt{\lambda_1} X\right)$$

$$\lambda_1 = j\omega^{\text{nd}} + 1$$

$$\tilde{C}_{-} = y_1 + y_2$$

$$y_2 = \beta_1 \sinh\left(\sqrt{\lambda_2} X\right) + \beta_2 \cosh\left(\sqrt{\lambda_2} X\right)$$

$$\lambda_2 = j\omega^{\text{nd}}$$

$$\tilde{C}_{+} = -\alpha_1 \sinh\left(\sqrt{\lambda_1} X\right) - \alpha_2 \cosh\left(\sqrt{\lambda_1} X\right) + \beta_1 \sinh\left(\sqrt{\lambda_2} X\right) + \beta_2 \cosh\left(\sqrt{\lambda_2} X\right)$$

$$\frac{\partial \tilde{C}_{+}}{\partial X} = -\alpha_1 \sqrt{\lambda_1} \cosh\left(\sqrt{\lambda_1} X\right) - \alpha_2 \sqrt{\lambda_1} \sinh\left(\sqrt{\lambda_1} X\right) + \beta_1 \sqrt{\lambda_2} \cosh\left(\sqrt{\lambda_2} X\right) + \beta_2 \sqrt{\lambda_2} \sinh\left(\sqrt{\lambda_2} X\right)$$

$$\tilde{C}_{-} = \alpha_1 \sinh\left(\sqrt{\lambda_1} X\right) + \alpha_2 \cosh\left(\sqrt{\lambda_1} X\right) + \beta_1 \sinh\left(\sqrt{\lambda_2} X\right) + \beta_2 \cosh\left(\sqrt{\lambda_2} X\right)$$

$$\frac{\partial \tilde{C}_{-}}{\partial X} = \alpha_1 \sqrt{\lambda_1} \cosh\left(\sqrt{\lambda_1} X\right) + \alpha_2 \sqrt{\lambda_1} \sinh\left(\sqrt{\lambda_1} X\right) + \beta_1 \sqrt{\lambda_2} \cosh\left(\sqrt{\lambda_2} X\right) + \beta_2 \sqrt{\lambda_2} \sinh\left(\sqrt{\lambda_2} X\right)$$

$$\frac{\partial \tilde{U}}{\partial X} = \frac{\alpha_1}{\sqrt{\lambda_1}} \cosh\left(\sqrt{\lambda_1} X\right) + \frac{\alpha_2}{\sqrt{\lambda_1}} \sinh\left(\sqrt{\lambda_1} X\right) + k_1$$

$$\tilde{U} = \frac{\alpha_1}{\lambda_1} \sinh\left(\sqrt{\lambda_1} X\right) + \frac{\alpha_2}{\lambda_1} \cosh\left(\sqrt{\lambda_1} X\right) + k_1 X + k_2$$

where,

$$\left.\frac{\partial \tilde{C}_{+}}{\partial X}\right|_{X=0} = -\alpha_1 \sqrt{\lambda_1} + \beta_1 \sqrt{\lambda_2} \qquad \left.\frac{\partial \tilde{C}_{-}}{\partial X}\right|_{X=0} = \alpha_1 \sqrt{\lambda_1} + \beta_1 \sqrt{\lambda_2}$$

$$\left.\frac{\partial \tilde{C}_{+}}{\partial X}\right|_{X=2\tilde{L}} = -\alpha_1 \sqrt{\lambda_1} \cosh\left(\sqrt{\lambda_1} 2\tilde{L}\right) - \alpha_2 \sqrt{\lambda_1} \sinh\left(\sqrt{\lambda_1} 2\tilde{L}\right) + \beta_1 \sqrt{\lambda_2} \cosh\left(\sqrt{\lambda_2} 2\tilde{L}\right) + \beta_2 \sqrt{\lambda_2} \sinh\left(\sqrt{\lambda_2} 2\tilde{L}\right)$$

$$\frac{\partial \tilde{C}_-}{\partial X}\bigg|_{X=2\tilde{L}} = \alpha_1\sqrt{\lambda_1}\cosh\left(\sqrt{\lambda_1}2\tilde{L}\right) + \alpha_2\sqrt{\lambda_1}\sinh\left(\sqrt{\lambda_1}2\tilde{L}\right) + \beta_1\sqrt{\lambda_2}\cosh\left(\sqrt{\lambda_2}2\tilde{L}\right) + \beta_2\sqrt{\lambda_2}\sinh\left(\sqrt{\lambda_2}2\tilde{L}\right)$$

$$\frac{\partial \widetilde{U}}{\partial X}\bigg|_{X=0} = \frac{\alpha_1}{\sqrt{\lambda_1}} + k_1 \qquad \frac{\partial \widetilde{U}}{\partial X}\bigg|_{X=\tilde{L}} = \frac{\alpha_1}{\sqrt{\lambda_1}}\cosh\left(\sqrt{\lambda_1}2\tilde{L}\right) + \frac{\alpha_2}{\sqrt{\lambda_1}}\sinh\left(\sqrt{\lambda_1}2\tilde{L}\right) + k_1$$

$$\widetilde{U}\Big|_{X=0} = \frac{\alpha_2}{\lambda_1} + k_2 \qquad \widetilde{U}\Big|_{X=\tilde{L}} = \frac{\alpha_1}{\lambda_1}\sinh\left(\sqrt{\lambda_1}2\tilde{L}\right) + \frac{\alpha_2}{\lambda_1}\cosh\left(\sqrt{\lambda_1}2\tilde{L}\right) + k_1\tilde{L} + k_2$$

where $\alpha_1, \alpha_2, \beta_1, \beta_2, k_1, k_2$ are determined by the boundary conditions.

For the DBCC, at $X = 0$, $\nabla\tilde{C}_+ + \nabla\widetilde{U} = 0$, we obtain,

$$-\alpha_1\sqrt{\lambda_1} + \beta_1\sqrt{\lambda_2} + \frac{\alpha_1}{\sqrt{\lambda_1}} + k_1 = 0,$$

and $\nabla\tilde{C}_- - \nabla\widetilde{U} = 0$, we obtain,

$$\alpha_1\sqrt{\lambda_1} + \beta_1\sqrt{\lambda_2} - \frac{\alpha_1}{\sqrt{\lambda_1}} - k_1 = 0.$$

Solving the above two equations, then we can obtain,

$$\beta_1 = 0,\ k_1 = \alpha_1\sqrt{\lambda_1} - \frac{\alpha_1}{\sqrt{\lambda_1}}.$$

And $\widetilde{U} = -\widetilde{U}_{\mathrm{M}} + r_c\nabla\widetilde{U}$ at $x = 0$, we can obtain,

$$\frac{\alpha_2}{\lambda_1} + k_2 = -\widetilde{U}_{\mathrm{M}} + r_c\left(\frac{\alpha_1}{\sqrt{\lambda_1}} + k_1\right),$$

with $r_c = \frac{C_{\mathrm{GC}}^0}{C_{\mathrm{H}}} = \frac{\delta_{\mathrm{H}}}{\lambda_D}\frac{\epsilon_{\mathrm{s}}}{\epsilon_{\mathrm{H}}}$, then we can obtain,

$$k_2 = -\widetilde{U}_{\mathrm{M}} + r_c\alpha_1\sqrt{\lambda_1} - \frac{\alpha_2}{\lambda_1},$$

At $x = 2\tilde{L}$, $\nabla\tilde{C}_+ + \nabla\widetilde{U} = 0$, we obtain,

$$-\alpha_1\sqrt{\lambda_1}\cosh\left(\sqrt{\lambda_1}2\tilde{L}\right) - \alpha_2\sqrt{\lambda_1}\sinh\left(\sqrt{\lambda_1}2\tilde{L}\right) + \beta_1\sqrt{\lambda_2}\cosh\left(\sqrt{\lambda_2}2\tilde{L}\right) + \beta_2\sqrt{\lambda_2}\sinh\left(\sqrt{\lambda_2}2\tilde{L}\right) + \frac{\alpha_1}{\sqrt{\lambda_1}}\cosh\left(\sqrt{\lambda_1}2\tilde{L}\right) + \frac{\alpha_2}{\sqrt{\lambda_1}}\sinh\left(\sqrt{\lambda_1}2\tilde{L}\right) + k_1 = 0,$$

and $\nabla\tilde{C}_- - \nabla\widetilde{U} = 0$, we obtain,

$$\alpha_1\sqrt{\lambda_1}\cosh\left(\sqrt{\lambda_1}2\tilde{L}\right)+\alpha_2\sqrt{\lambda_1}\sinh\left(\sqrt{\lambda_1}2\tilde{L}\right)+\beta_1\sqrt{\lambda_2}\cosh\left(\sqrt{\lambda_2}2\tilde{L}\right)+\beta_2\sqrt{\lambda_2}\sinh\left(\sqrt{\lambda_2}2\tilde{L}\right)-\frac{\alpha_1}{\sqrt{\lambda_1}}\cosh\left(\sqrt{\lambda_1}2\tilde{L}\right)-\frac{\alpha_2}{\sqrt{\lambda_1}}\sinh\left(\sqrt{\lambda_1}2\tilde{L}\right)-k_1=0,$$

Then we can obtain

$$\alpha_1=\tilde{U}_{\mathrm{M}}\frac{\sinh\left(\sqrt{\lambda_1}2\tilde{L}\right)}{\left(2r_c\sqrt{\lambda_1}+2\tilde{L}\sqrt{\lambda_1}-\frac{2\tilde{L}}{\sqrt{\lambda_1}}\right)\sinh\left(\sqrt{\lambda_1}2\tilde{L}\right)+\frac{2}{\lambda_1}\left(\cosh\left(\sqrt{\lambda_1}2\tilde{L}\right)-1\right)},$$

$$\alpha_2=\alpha_1\frac{1-\cosh\left(\sqrt{\lambda_1}2\tilde{L}\right)}{\sinh\left(\sqrt{\lambda_1}2\tilde{L}\right)}=\tilde{U}_{\mathrm{M}}\frac{1-\cosh\left(\sqrt{\lambda_1}2\tilde{L}\right)}{\left(2r_c\sqrt{\lambda_1}+2\tilde{L}\sqrt{\lambda_1}-\frac{2\tilde{L}}{\sqrt{\lambda_1}}\right)\sinh\left(\sqrt{\lambda_1}2\tilde{L}\right)+\frac{2}{\lambda_1}\left(\cosh\left(\sqrt{\lambda_1}2\tilde{L}\right)-1\right)}.$$

when the EDL charge refers to $\tilde{Q}_{\mathrm{EDL}}$,

$$\begin{aligned}
\tilde{J}_{\mathrm{EDL}}&=-j\omega^{\mathrm{nd}}\int_0^{\tilde{L}}\left(\tilde{C}_+-\tilde{C}_-\right)dX\\
&=2j\omega^{\mathrm{nd}}\int_0^{\tilde{L}}\left(\alpha_1\sinh\left(\sqrt{\lambda_1}X\right)+\alpha_2\cosh\left(\sqrt{\lambda_1}X\right)\right)dX\\
&=2j\omega^{\mathrm{nd}}\left(\frac{\alpha_1}{\sqrt{\lambda_1}}\cosh\left(\sqrt{\lambda_1}X\right)+\frac{\alpha_2}{\sqrt{\lambda_1}}\sinh\left(\sqrt{\lambda_1}X\right)\right)\Bigg|_0^{\tilde{L}}\\
&=2j\omega^{\mathrm{nd}}\left(\frac{\alpha_1}{\sqrt{\lambda_1}}\cosh\left(\sqrt{\lambda_1}\tilde{L}\right)+\frac{\alpha_2}{\sqrt{\lambda_1}}\sinh\left(\sqrt{\lambda_1}\tilde{L}\right)-\frac{\alpha_1}{\sqrt{\lambda_1}}\right) \qquad \text{(S11)}\\
&=2j\omega^{\mathrm{nd}}\left(\tilde{U}_{\mathrm{M}}\frac{\sinh\left(\sqrt{\lambda_1}2\tilde{L}\right)}{\mathrm{A}}\frac{1}{\sqrt{\lambda_1}}\cosh\left(\sqrt{\lambda_1}\tilde{L}\right)\right.\\
&\quad+\tilde{U}_{\mathrm{M}}\frac{1-\cosh\left(\sqrt{\lambda_1}2\tilde{L}\right)}{\mathrm{A}}\frac{1}{\sqrt{\lambda_1}}\sinh\left(\sqrt{\lambda_1}\tilde{L}\right)\\
&\quad\left.-\tilde{U}_{\mathrm{M}}\frac{\sinh\left(\sqrt{\lambda_1}2\tilde{L}\right)}{\mathrm{A}}\frac{1}{\sqrt{\lambda_1}}\right)
\end{aligned}$$

with

$$\mathrm{A}=\left(2r_c\sqrt{\lambda_1}+2\tilde{L}\sqrt{\lambda_1}-\frac{2\tilde{L}}{\sqrt{\lambda_1}}\right)\sinh\left(\sqrt{\lambda_1}2\tilde{L}\right)+\frac{2}{\lambda_1}\left(\cosh\left(\sqrt{\lambda_1}2\tilde{L}\right)-1\right)$$

and the impedance is calculated,

$$Z_{\mathrm{EDL}}^{\mathrm{nd}} = \frac{\widetilde{U}_{\mathrm{M}}}{\tilde{J}_{\mathrm{EDL}}}$$

$$= \frac{1}{2j\omega^{\mathrm{nd}}\left(\frac{\sinh\left(\sqrt{\lambda_1}2\tilde{L}\right)}{\mathrm{A}}\frac{1}{\sqrt{\lambda_1}}\cosh\left(\sqrt{\lambda_1}\tilde{L}\right) + \frac{1-\cosh\left(\sqrt{\lambda_1}2\tilde{L}\right)}{\mathrm{A}}\frac{1}{\sqrt{\lambda_1}}\sinh\left(\sqrt{\lambda_1}\tilde{L}\right) - \frac{\sinh\left(\sqrt{\lambda_1}2\tilde{L}\right)}{\mathrm{A}}\frac{1}{\sqrt{\lambda_1}}\right)}$$

$$= \frac{1}{2j\omega^{\mathrm{nd}}}\frac{\mathrm{A}\sqrt{\lambda_1}}{\left(1-\cosh\left(\sqrt{\lambda_1}2\tilde{L}\right)\right)\sinh\left(\sqrt{\lambda_1}\tilde{L}\right) - \left(1-\cosh\left(\sqrt{\lambda_1}\tilde{L}\right)\right)\sinh\left(\sqrt{\lambda_1}2\tilde{L}\right)} \tag{S12}$$

$$= \frac{1}{2j\omega^{\mathrm{nd}}}\frac{\left(2r_c\lambda_1 + 2\tilde{L}\lambda_1 - 2\tilde{L}\right)\tanh\left(\sqrt{\lambda_1}2\tilde{L}\right) + \frac{2}{\sqrt{\lambda_1}}\left(1-\mathrm{sech}\left(\sqrt{\lambda_1}2\tilde{L}\right)\right)}{\left(\mathrm{sech}\left(\sqrt{\lambda_1}2\tilde{L}\right)-1\right)\sinh\left(\sqrt{\lambda_1}\tilde{L}\right) - \left(1-\cosh\left(\sqrt{\lambda_1}\tilde{L}\right)\right)\tanh\left(\sqrt{\lambda_1}2\tilde{L}\right)}$$

When the EDL charge refers to $\tilde{Q}_{\mathrm{M}}$,

$$\tilde{J}_{\mathrm{M}} = j\omega^{\mathrm{nd}}\frac{D}{\lambda_D^2}\frac{\epsilon_{\mathrm{S}}RT}{F^2c_0D}\frac{dU}{dX}\bigg|_{x\,=\,0^+} = 2j\omega^{nd}\frac{dU}{dX}\bigg|_{X\,=\,0^+}$$

$$= 2j\omega^{\mathrm{nd}}\left(\frac{\alpha_1}{\sqrt{\lambda_1}} + \alpha_1\sqrt{\lambda_1} - \frac{\alpha_1}{\sqrt{\lambda_1}}\right) = 2j\omega^{nd}\alpha_1\sqrt{\lambda_1} \tag{S13}$$

$$= 2j\omega^{\mathrm{nd}}\widetilde{U}_{\mathrm{M}}\frac{\sinh\left(\sqrt{\lambda_1}2\tilde{L}\right)}{\left(2r_c\sqrt{\lambda_1} + 2\tilde{L}\sqrt{\lambda_1} - \frac{2\tilde{L}}{\sqrt{\lambda_1}}\right)\sinh\left(\sqrt{\lambda_1}2\tilde{L}\right) + \frac{2}{\lambda_1}\left(\cosh\left(\sqrt{\lambda_1}2\tilde{L}\right)-1\right)}\sqrt{\lambda_1}$$

and the impedance is calculated,

$$Z_{\mathrm{M}}^{\mathrm{nd}} = \frac{\widetilde{U}_{\mathrm{M}}}{\tilde{J}_{\mathrm{M}}}$$

$$= \frac{1}{2j\omega^{\mathrm{nd}}}\frac{\left(2r_c\sqrt{\lambda_1} + 2\tilde{L}\sqrt{\lambda_1} - \frac{2\tilde{L}}{\sqrt{\lambda_1}}\right)\sinh\left(\sqrt{\lambda_1}2\tilde{L}\right) + \frac{2}{\lambda_1}\left(\cosh\left(\sqrt{\lambda_1}2\tilde{L}\right)-1\right)}{\sqrt{\lambda_1}\sinh\left(\sqrt{\lambda_1}2\tilde{L}\right)} \tag{S14}$$

$$= \frac{1}{2j\omega^{\mathrm{nd}}}\frac{\left(2r_c\lambda_1 + 2\tilde{L}\lambda_1 - 2\tilde{L}\right)\sinh\left(\sqrt{\lambda_1}2\tilde{L}\right) + \frac{2}{\sqrt{\lambda_1}}\left(\cosh\left(\sqrt{\lambda_1}2\tilde{L}\right)-1\right)}{\lambda_1\sinh\left(\sqrt{\lambda_1}2\tilde{L}\right)}$$

For the SBOC, in the similar way, we can obtain,

$$\alpha_1 = -\frac{\widetilde{U}_{\mathrm{M}}}{r_c\sqrt{\lambda_1} + \frac{\tanh\left(\sqrt{\lambda_1}\tilde{L}\right)}{\lambda_1} + \tilde{L}\left(\sqrt{\lambda_1} - \frac{1}{\sqrt{\lambda_1}}\right)}$$

$$\alpha_2 = \frac{\widetilde{U}_{\mathrm{M}}\tanh\left(\sqrt{\lambda_1}\tilde{L}\right)}{r_c\sqrt{\lambda_1} + \frac{\tanh\left(\sqrt{\lambda_1}\tilde{L}\right)}{\lambda_1} + \tilde{L}\left(\sqrt{\lambda_1} - \frac{1}{\sqrt{\lambda_1}}\right)}$$

$$\beta_1 = \beta_2 = 0$$

$$k_1 = \left(\sqrt{\lambda_1} - \frac{1}{\sqrt{\lambda_1}}\right)\alpha_1$$

$$k_2 = -\left(\sqrt{\lambda_1} - \frac{1}{\sqrt{\lambda_1}}\right)\alpha_1 L$$

$$\frac{\partial\widetilde{U}}{\partial X} = \frac{\alpha_1}{\sqrt{\lambda_1}}\cosh\left(\sqrt{\lambda_1}X\right) + \frac{\alpha_2}{\sqrt{\lambda_1}}\sinh\left(\sqrt{\lambda_1}X\right) + k_1$$

When the EDL charge refers to $\tilde{Q}_{\mathrm{EDL}}$, the impedance is calculated,

$$Z_{\mathrm{EDL}}^{\mathrm{nd}} = \frac{\widetilde{U}_{\mathrm{M}}}{\tilde{J}_{\mathrm{EDL}}} = \frac{1}{2j\omega^{\mathrm{nd}}}\frac{r_c\lambda_1 + \frac{\tanh\left(\sqrt{\lambda_1}\tilde{L}\right)}{\sqrt{\lambda_1}} + \tilde{L}(\lambda_1 - 1)}{1 - \mathrm{sech}\left(\sqrt{\lambda_1}\tilde{L}\right)} \tag{S15}$$

When the EDL charge refers to $\tilde{Q}_{\mathrm{M}}$, and the impedance is calculated,

$$Z_{\mathrm{M}}^{\mathrm{nd}} = \frac{\widetilde{U}_{\mathrm{M}}}{\tilde{J}_{\mathrm{EDL}}} = \frac{1}{2j\omega^{\mathrm{nd}}}\frac{r_c\lambda_1 + \frac{\tanh\left(\sqrt{\lambda_1}\tilde{L}\right)}{\sqrt{\lambda_1}} + \tilde{L}(\lambda_1 - 1)}{\lambda_1} \tag{S16}$$

where Z^{nd} is the dimensionless form with respect to $\frac{2\lambda_D^2}{D_+ C_{GC}}$, $r_{\mathrm{c}} = C_{\mathrm{GC}}^0/C_{\mathrm{H}}$ the ratio between the Gouy-Chapman capacitance at PZC, $C_{\mathrm{GC}}^0 = \frac{\epsilon_s}{\lambda_D}$ and the Helmholtz capacitance, $C_{\mathrm{H}} = \frac{\epsilon_{\mathrm{HP}}}{\delta_{\mathrm{HP}}}$, and $\omega^{nd} = \omega\frac{\lambda_D^2}{D_+}$. Take the SBOC for example, whose dimensional form is expressed as,

$$Z_{\mathrm{EDL}} = \frac{1}{j\omega C_{\mathrm{H}}}\frac{\lambda_1}{1 - \mathrm{sech}\left(\sqrt{\lambda_1}L/\lambda_D\right)} + \frac{1}{j\omega C_{\mathrm{GC}}^0}\frac{\frac{\tanh\left(\sqrt{\lambda_1}L/\lambda_D\right)}{\sqrt{\lambda_1}} + \frac{L}{\lambda_D}(\lambda_1 - 1)}{1 - \mathrm{sech}\left(\sqrt{\lambda_1}L/\lambda_D\right)}. \tag{S17}$$

In the low frequency range, namely, $\omega \to 0$, $\lambda_1 \approx 1$, $1 - \mathrm{sech}(L/\lambda_D) \approx 1$, and

$\tanh(L/\lambda_D) \approx 1$. Then Eq. (S17) is asymptotic to

$$Z_{\mathrm{EDL}} = \frac{1}{j\omega}\left(\frac{1}{C_{\mathrm{H}}} + \frac{1}{C_{\mathrm{GC}}^{0}}\right), \tag{S18}$$

a capacitive behavior corresponding to the equilibrium EDL capacitance. In the high frequency range, namely, $\omega \to \infty$, $\lambda_1 \approx j\omega \frac{\lambda_D^2}{D_+}$, $1 - \mathrm{sech}\left(\sqrt{\lambda_1}L/\lambda_D\right) \approx 1$, $\lambda_1 - 1 \approx \lambda_1$, and $\frac{\tanh\left(\sqrt{\lambda_1}L/\lambda_D\right)}{\sqrt{\lambda_1}} \approx 0$. Then Eq. (S17) is asymptotic to,

$$Z_{\mathrm{EDL}} = \frac{\lambda_D^2}{C_{\mathrm{H}}D_+} + \frac{\lambda_D L}{C_{\mathrm{GC}}^{0}D_+} \approx \frac{L}{\frac{2F^2c_0D_+}{RT}} = \frac{L}{\sigma_s}, \tag{S19}$$

a pure resistance behavior, where $\sigma_{\mathrm{s}} = \frac{2F^2c_0D_+}{RT}$ is the electrical conductivity of the bulk electrolyte.

When the EDL charge refers to the metal surface charge, the impedance reads,

$$Z_{\mathrm{M}} = \frac{1}{j\omega C_{\mathrm{H}}} + \frac{1}{j\omega C_{\mathrm{GC}}^{0}} \frac{\frac{\tanh\left(\sqrt{\lambda_1}L/\lambda_D\right)}{\sqrt{\lambda_1}} + \frac{L}{\lambda_D}(\lambda_1 - 1)}{\lambda_1}. \tag{S20}$$

In the low frequency range, namely, $\omega \to 0$, $\lambda_1 \approx 1$, and $\tanh(L/\lambda_D) \approx 1$. Then Eq. (S20) is asymptotic to

$$Z_{\mathrm{M}} = \frac{1}{j\omega}\left(\frac{1}{C_{\mathrm{H}}} + \frac{1}{C_{\mathrm{GC}}^{0}}\right), \tag{S21}$$

which is the same as Z_{EDL} in the low frequency range. In the high frequency range, namely, $\omega \to \infty$, $\frac{\tanh\left(\sqrt{\lambda_1}L/\lambda_D\right)}{\sqrt{\lambda_1}} \approx 0$. Then Eq. (S20) is asymptotic to,

$$Z_{\mathrm{M}} = \frac{1}{j\omega C_{\mathrm{H}}} + \frac{1}{j\omega C_{\mathrm{GC}}^{0}} \frac{1}{\frac{\lambda_D}{L}\left(1 + \frac{D_+}{j\omega\lambda_D^2}\right)} = \frac{1}{j\omega C_{\mathrm{H}}} + \frac{1}{\frac{C_{\mathrm{GC}}^{0}D_+}{\lambda_D L} + j\omega\frac{\epsilon_{\mathrm{s}}}{L}} \approx \frac{1}{\frac{1}{R_{\mathrm{ele}}^{\mathrm{pzc}}} + j\omega C_{\mathrm{geo}}}, \tag{S22}$$

where $R_{\mathrm{ele}}^{\mathrm{pzc}} = \frac{\lambda_D L}{C_{\mathrm{GC}}^{0}D_+} = \frac{L}{\sigma_{\mathrm{s}}}$ is the electrolyte resistance at the PZC and $C_{\mathrm{geo}} = \frac{\epsilon_{\mathrm{s}}}{L}$ the geometric capacitance of the electrolyte.

7. Comparison of EIS between linear PNP and nonlinear PNP theory for the SBOC

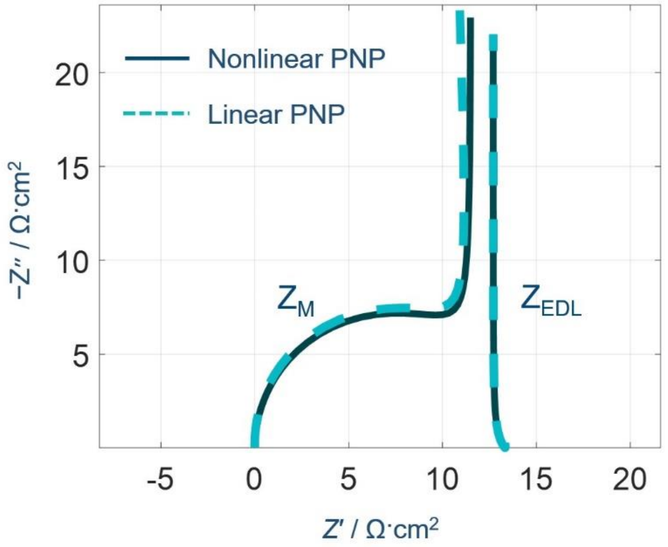

FIG S7. For the SBOC, at the potential of zero charge, we compare numerical EIS results between the nonlinear PNP (solid line) and linear PNP theory (dashed line). Model parameters are: $L = 100\ \mathrm{nm}, U_\mathrm{M}^\mathrm{dc} = 0,\ c_0 = 1 \times 10^{-3}\ \mathrm{mol\ L^{-1}}, D_\pm = 1 \times 10^{-11}\ \mathrm{m^2\ s^{-1}}, \delta_\mathrm{HP} = 0\ \mathrm{nm}$.

8. EIS response of the DBCC

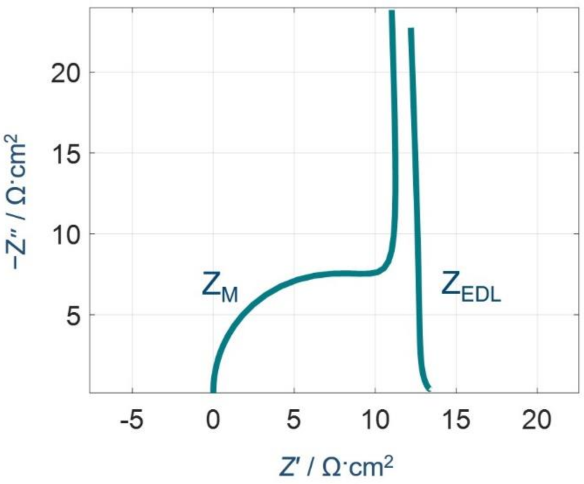

FIG S8. For the DBCC, comparison between the EIS calculated from the total diffuse charge, denoted as Z_{EDL}, and that from the electrode surface charge, Z_{M}, at the potential of zero charge, $U_{\mathrm{M}}^{\mathrm{dc}} = 0$. Model parameters are $c_0 = 1 \times 10^{-3}\ \mathrm{mol\ L^{-1}}$, $D_{\pm} = 1 \times 10^{-11}\ \mathrm{m^2\ s^{-1}}$, $\delta_{\mathrm{HP}} = 0\ \mathrm{nm}$, $E_{\mathrm{M}} = 2.5 \times 10^{-3} \sin \omega t\ \ \mathrm{V}$, $E_{\mathrm{eq}} = E_{\mathrm{pzc}} = 0$. Frequency range, $1 \times 10^{6} \sim 1 \times 10^{-1}$ Hz.

9. EIS response of the single blocking closed cell

We consider a case with a non-blocking metal on the right side, namely, the single-blocking closed cell, as shown in FIG S9(a). A charging transfer occurs at the $X = L$,

$$\mathrm{M} \leftrightarrow \mathrm{M}^{+} + \mathrm{e}^{-} \tag{S23}$$

where M^{+} is the metal ion, and M the metal atom. This charge transfer is described using the FBV theory,

$$j_{ct} = k_{0,ct}\left(\exp\left(\frac{\alpha F\eta}{RT}\right) - C_{+,\mathrm{L}}\exp\left(-\frac{(1-\alpha)F\eta}{RT}\right)\right) \tag{S24}$$

where $k_{0,rct}$ is the rate constant of charge transfer, $C_{+,\mathrm{L}}$ the concentration of M^{+} at the right boundary, α the charge transfer coefficient and $\eta = -E_{\mathrm{M}} - \phi_{\mathrm{HP}} - E_{\mathrm{eq}}$ the overpotential with E_{eq} being the equilibrium potential and $-E_{\mathrm{M}}$ the electrode potential at the right electrode. The other conditions are the same as those in the SBOC case. FIG S9(b) compares the EIS numerically calculated at different $k_{0,ct}$ at the potential of zero charge, $U_{\mathrm{M}}^{\mathrm{dc}} = 0, L = 100$ nm, $\delta_{\mathrm{HP}} = 0$ nm. We notice that the EIS consists of two semicircles in high and intermediate frequency range, respectively, and a vertical line in low frequency range. With increasing $k_{0,ct}$, the intermediate-frequency semicircle associated with the charging transfer decreases. The high-frequency semicircle corresponds to the electrolyte resistance in parallel with the geometric capacitance, and the low-frequency vertical line corresponds to the equilibrium EDL capacitance.

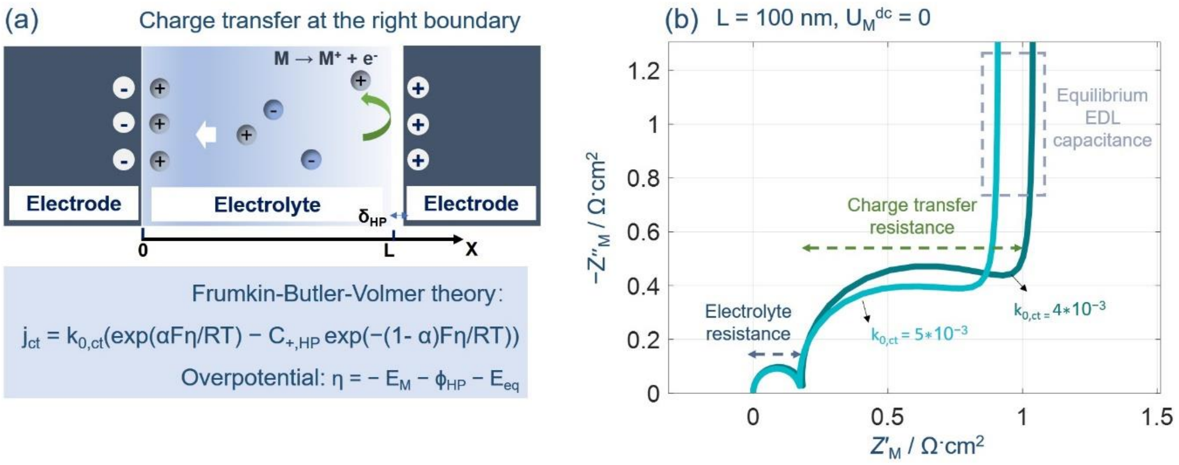

FIG S9. (a) Schematic diagram of the single blocking closed cell with one side in contact with a blocking electrode, and the other side connected to a non-blocking electrode; (b) The EIS of single blocking closed cell at different rate constant of charge transfer $k_{0,ct}$, at the potential of zero charge, $U_{\mathrm{M}}^{\mathrm{dc}} = 0, L = 100\ \mathrm{nm}, \delta_{\mathrm{HP}} = 0.3\ \mathrm{nm}$. Model parameters are $c_0 = 0.1\ \mathrm{mol\ L^{-1}}$, $D_{\pm} = 5 \times 10^{-12}\ \mathrm{m^2\ s^{-1}}$, $E_{\mathrm{M}} = 2.5 \times 10^{-3} \sin \omega t\ \ \mathrm{V}$, $E_{\mathrm{eq}} = E_{\mathrm{pzc}} = 0$. Frequency range is $1 \times 10^{7} \sim 1 \times 10^{-1}$ Hz.

Reference

1. Jiang, J.; Cao, D.; Jiang, D. E.; Wu, J, Kinetic Charging Inversion in Ionic Liquid Electric Double Layers. *The journal of physical chemistry letters* **2014,** 5, (13), 2195-200.

2. Ma, K.; Janssen, M.; Lian, C.; van Roij, R, Dynamic density functional theory for the charging of electric double layer capacitors. *The Journal of Chemical Physics* **2022,** 156, (8), 084101.

3. Lopez-Garcia, J. J.; Horno, J.; Grosse, C, Impedance-Frequency Response of Closed Electrolytic Cells. *Micromachines* **2023,** 14, (2), 368.

4. Li, C. K., Huang, J, Impedance Response of Electrochemical Interfaces: Part I. Exact Analytical Expressions for Ideally Polarizable Electrodes. *Journal of The Electrochemical Society* **2021,** 167, (16), 166517.

5. Zhang, L. L., Li, C. K., Huang, J, A Beginners' Guide to Modelling of Electric Double Layer under Equilibrium, Nonequilibrium and AC Conditions. *Journal of Electrochemistry* **2022,** 28, (2), 2108471.
